# Statistical analysis plan for the TRANSLATE (TRANSrectal biopsy versus Local Anaesthetic Transperineal biopsy Evaluation of potentially clinically significant prostate cancer) multicentre randomised controlled trial

**DOI:** 10.1186/s13063-024-08224-4

**Published:** 2024-06-14

**Authors:** Ioana R. Marian, Alexander Ooms, Jane Holmes, Matthew J. Parkes, Alastair D. Lamb, Richard J. Bryant

**Affiliations:** 1 Oxford Clinical Trials Research Unit, University of Oxford, Botnar Research Centre, Old Road OX3 7LD, Oxford, UK; 2 Nuffield Department of Primary Care Health Sciences, University of Oxford, Oxford, UK; 3 Centre for Biostatistics, Division of Population Health, Health Services Research & Primary Care, School of Health Sciences, The University of Manchester, Manchester, UK; 4 NIHR Manchester Biomedical Research Centre, Manchester University NHS Foundation Trust, Manchester Academic Health Science Centre (MAHSC), Manchester, UK; 5 Department of Urology, Oxford University Hospitals NHS Foundation Trust, Churchill Hospital, Oxford, UK; 6 Nuffield Department of Surgical Sciences, University of Oxford, Oxford, UK

**Keywords:** Statistical analysis plan, Randomised controlled trial, Prostate cancer, Cancer biopsy, Cancer diagnosis

## Abstract

**Background:**

The TRANSLATE (TRANSrectal biopsy versus Local Anaesthetic Transperineal biopsy Evaluation) trial assesses the clinical and cost-effectiveness of two biopsy procedures in terms of detection of clinically significant prostate cancer (PCa). This article describes the statistical analysis plan (SAP) for the TRANSLATE randomised controlled trial (RCT).

**Methods/design:**

TRANSLATE is a parallel, superiority, multicentre RCT. Biopsy-naïve men aged ≥ 18 years requiring a prostate biopsy for suspicion of possible PCa are randomised (computer-generated 1:1 allocation ratio) to one of two biopsy procedures: transrectal (TRUS) or local anaesthetic transperineal (LATP) biopsy. The primary outcome is the difference in detection rates of clinically significant PCa (defined as Gleason Grade Group ≥ 2, i.e. any Gleason pattern ≥ 4 disease) between the two biopsy procedures. Secondary outcome measures are th eProBE questionnaire (Perception Part and General Symptoms) and International Index of Erectile Function (IIEF, Domain A) scores, International Prostate Symptom Score (IPSS) values, EQ-5D-5L scores, resource use, infection rates, complications, and serious adverse events. We describe in detail the sample size calculation, statistical models used for the analysis, handling of missing data, and planned sensitivity and subgroup analyses. This SAP was pre-specified, written and submitted without prior knowledge of the trial results.

**Discussion:**

Publication of the TRANSLATE trial SAP aims to increase the transparency of the data analysis and reduce the risk of outcome reporting bias. Any deviations from the current SAP will be described and justified in the final study report and results publication.

**Trial registration:**

International Standard Randomised Controlled Trial Number ISRCTN98159689, registered on 28 January 2021 and registered on the ClinicalTrials.gov (NCT05179694) trials registry.

**Supplementary Information:**

The online version contains supplementary material available at 10.1186/s13063-024-08224-4.

## Background

Prostate cancer (PCa) has been diagnosed over the last several decades through a procedure termed a “transrectal ultrasound-guided” (TRUS) biopsy. During a TRUS biopsy, an ultrasound imaging probe is placed in the rectum and through this a biopsy needle is inserted into the prostate gland to take prostate tissue biopsy samples. Evidence shows that TRUS biopsies can miss detection of approximately one third of significant PCa cases, and have a risk of causing severe infection as the biopsy needle passes through the rectum [1]. An alternative option is to take prostate biopsy samples through perineal skin under local anaesthetic, known as a “local anaesthetic transperineal prostate” (LATP) biopsy. By allowing the needle to be directed into the prostate gland via the transperineal approach, LATP may be better at detecting clinically significant PCa, with a lower risk of infection.

TRANSrectal biopsy versus Local Anaesthetic Transperineal biopsy Evaluation (TRANSLATE) is a parallel group design randomised controlled trial (RCT) assessing two biopsy procedures: TRUS, routinely used in diagnostic centres for men with suspected PCa, and an alternative more recent method, LATP, chosen to replace TRUS in some individual centres despite the absence of level one evidence that it is superior to TRUS. The aim of this study is to determine whether LATP biopsies are better than TRUS at detecting clinically significant PCa. We will also investigate the tolerability of the two biopsies, along with infection and complication rates, admissions to hospital, need for a repeat biopsy procedure, and whether the use of LATP biopsy is more cost-effective compared to TRUS biopsy.

This article reports details of the pre-specified statistical analysis plan (SAP) prepared according to the published guidelines on the content of SAPs [2] and reviewed by the Data, Safety and Monitoring Committee (DSMC) and Trial Steering Committee (TSC) in August 2023. Full details of the trial design, study population and study procedures are available in the published TRANSLATE protocol [3]. Details of the Health Economics analyses are described in the study protocol [3] and will be undertaken separately by a Health Economist.

## Methods and design

### Trial design

TRANSLATE is a parallel-group, superiority, multicentre RCT comparing LATP against TRUS biopsy, with respect to the detection of clinically significant PCa. The study features two stages: the first stage is a randomised internal pilot which aims to demonstrate the feasibility of identifying and randomising men with a suspicion of PCa; the second stage comprises the full definitive trial, aiming to assess the effectiveness of LATP versus TRUS biopsy in diagnosing clinically significant PCa, whilst additionally investigating the two biopsy techniques in terms of their risk of significant infection-related and other complications, tolerability, impact on quality of life, and cost-effectiveness.

#### Randomisation

Eligible men who consent to join TRANSLATE are randomised to one of the two interventions in a 1:1 ratio using minimisation, minimised by recruiting centre and magnetic resonance imaging (MRI) lesion location (i.e. ‘No significant lesion’, ‘Significant lesion, including anterior’, ‘Significant lesion, but not anterior’). The minimisation algorithm includes a probabilistic element, and a small number of participants are initially randomised by simple randomisation, to ensure the unpredictability of biopsy allocation. Minimisation by the recruitment centre will help ensure that any centre effect will be equally distributed in the trial arms, and enable potential practical issues associated with biopsy delivery or site closure to be accounted for. A minimum of nine urological centres across the UK will participate in the study. There is some evidence that MRI lesion location can affect PCa detection, therefore it is important for the two prostate biopsy techniques to be balanced across this potentially important prognostic factor. The randomisation is performed via a secure centralised web-based system (“Registration/Randomisation and Management of Product”) via the REDCap (Research Electronic Data Capture) database interface at the Oxford Clinical Trials Research Unit (OCTRU), consistent with UK Clinical Research Collaboration-approved standard operating procedures [4, 5]. This system ensures prospective registration and allocation concealment until the point at which the patient enters the trial.

#### Eligibility

Potential participants are eligible for the TRANSLATE trial if they are biopsy-naïve men aged ≥ 18 years who, during investigation for suspicion of possible PCa, require a prostate biopsy, with a prostate-specific antigen (PSA) value above the age-adjusted upper limit of normal regardless of the pre-biopsy MRI result, *or* an abnormal pre-biopsy MRI on a 1.5 T or higher MRI scanner, *or* an abnormal prostate digital rectal examination (DRE) (regardless of serum PSA or MRI result). All participants are considered suitable to tolerate a LATP biopsy procedure by the local clinical team, able to give informed consent, and able to understand written English. The following patients will not be able to join the study: any individuals with a previous prostate biopsy; dysuria on the day of biopsy or untreated urinary tract infection (UTI); immunocompromised (due to a history of a prior immunocompromising medical condition, or medications e.g. steroids or methotrexate); those who may need enhanced antibiotic prophylaxis (such as those with an indwelling catheter, or who have recurrent UTIs); previous abdomino-perineal resection (i.e. absent rectum); unable to recline adequately in Lloyd-Davis/lithotomy position (e.g. hip surgery, contractures); unable to have a pre-biopsy MRI (e.g. pacemaker, estimated glomerular filtration rate (eGFR) < 50); PSA > 50 ng/ml (> 25 ng/ml if the patient is on finasteride) (to exclude patients with locally advanced/metastatic PCa, easily detectable by either biopsy technique).

#### Blinding

The study participants and clinical team delivering the biopsy cannot be blinded to the allocated procedure due to the nature of the intervention. The Chief Investigators will remain blinded to treatment allocation for the overall clinical trial participants (i.e. knowledge of treatment allocation is limited to those participants at the Chief Investigators’ own site). In instances where serious adverse events (SAEs) are reported, the Chief Investigators will be unblinded to complete a full causality assessment. The DSMC and TSC will be blinded to biopsy allocation when reviewing data. The trial statistician and data entry personnel are not blinded to allocation. The trial statistician will perform a blinded assessment of data (not separated by intervention group) before the final data analysis and any pre-specification of analyses will be written before without prior knowledge of the trial results. The remaining members of the trial management team are blinded to allocation until the completion of data analysis.

### Objectives

The primary objective of the TRANSLATE trial is to compare TRUS biopsy versus LATP biopsy in the detection of clinically significant PCa (defined as Gleason Grade Group (GGG) ≥ 2, i.e. any Gleason pattern ≥ 4 disease) assessed immediately after the biopsy procedure.

Secondary objectives are to investigate if there are any differences between the two biopsy techniques in the following:

- Rates of infection (7 days, 35 days and 4 months post-procedure)
- Participant reported tolerability of the procedure (immediately post-procedure)
- Participant reported biopsy-related complications (7 days, 35 days and 4 months post-procedure)
- Quality of Life (7 days, 35 days and 4 months post-procedure)
- Histological parameters, including GGG ≥ 3 PCa (immediately post-procedure)
- Number of subsequent prostate biopsy procedures required and details of the subsequent biopsies (35 days and 4 months post-procedure)
- Burden and rate of detection of clinically insignificant (GGG1) PCa (immediately post-procedure)
- SAE incidence (7 days, 35 days and 4 months post-procedure).

### Outcomes

#### Primary outcome

The primary outcome is the difference in the detection rate of clinically significant PCa upon pathology reporting of biopsy samples between the TRUS and LATP biopsy groups, the clinically significant disease being defined as a GGG (ISUP) score of 2 or more, i.e., any Gleason Pattern ≥ 4 disease [6]. The GGG score is a pathology-based endpoint and is usually available within 7 days of the initial biopsy, although difficult cases or other pathway delays may result in a longer period.

#### Secondary outcomes

The secondary outcome measures assessed in TRANSLATE and associated time points are described in Table 1 and presented in detail below.

**Table 1:** Time points at which outcomes are assessed

Time point	Outcome measure
Baseline	Participant demographics (Charlson Comorbidity Index, cancer history, DRE, PSA, current medications, body mass index, ethnicity) IIEF questionnaire (Domain A) I-PSS questionnaire EQ-5D-5L questionnaire
Immediately post-procedure	ProBE questionnaire (Perception part) Histological parameters (ISUP grade group^a^, cancer core length, core involvement, target biopsy cancer parameters) Burden and rate of detection of clinically significant (GGG ≥ 2) PCa Burden and rate of detection of GGG ≥ 3 PCa Burden and rate of detection of clinically insignificant (GGG 1) PCa
7 days post-procedure	ProBE questionnaire (General Symptoms part) IIEF questionnaire (Domain A) I-PSS questionnaire EQ-5D-5L questionnaire Infection Complications and SAEs
35 days post-procedure	IIEF questionnaire (Domain A) I-PSS questionnaire EQ-5D-5L questionnaire Infection Complications and SAEs Number of subsequent prostate biopsy procedures
4 months post-procedure	IIEF questionnaire (Domain A) I-PSS questionnaire EQ-5D-5L questionnaire Infection Complications and SAEs Number of subsequent prostate biopsy procedures and details of the subsequent biopsies

^a^Includes the primary outcome measure, the difference in detection rates of clinically significant PCa, defined as Gleason Grade Group ≥ 2, i.e. any Gleason pattern ≥ 4 disease

The *International Index of Erectile Function (IIEF) Domain A* [7] is a participant-reported questionnaire with six questions that examine male erectile function. Each question is marked from 0 to 5, with a total score range between 0 and 30, where lower values represent worse outcomes.

The *International Prostate Symptom Score (I-PSS)* [8] consists of seven sections concerning urinary symptoms, each scored on a scale from 0 to 5 (0 is “not at all” and 5 is “almost always”). The total I-PSS score ranges from 0 to 35, with higher scores indicative of more severe symptoms. The seven subsections are “incomplete emptying”, “frequency”, “intermittency”, “urgency”, “weak stream”, “straining” and “nocturia”. The I-PSS questionnaire also includes a “quality of life (QoL) due to urinary symptoms” measure with a score from 0 to 6 where 0 means “delighted” and 6 is “terrible”.

The *EQ-5D-5L* is a generic health-related QoL measure consisting of 5 dimensions each with a 5-level answer possibility and a health thermometer visual analogue scale (EQ VAS) [9, 10]. The EQ-5D-5L will be converted into a health utility score where 1 represents perfect health and 0 indicates health states equal to death, to report health-related QoL [11]. Negative values on the utility scale are possible. The VAS takes values between 0 and 100, where 0 represents worst imaginable health and 100 best imaginable health.

*Participant-reported tolerability* of the prostate biopsy procedure will be measured using the *Prostate Biopsy Effects* (ProBE) questionnaire (Perception part) [12] completed by the participant immediately after the procedure. Participants will report biopsy-related *complications* (such as bleeding, pain, urinary retention) as part of the *Resource Use* questionnaire at all follow-up timepoints and using the *ProBE* questionnaire *(General Symptoms part)* at 7 days after the procedure [12].

*Infection* in this trial is defined as any symptoms or signs of infection resulting in hospitalisation as indicated by data collected from patient notes and reported by the clinicians throughout the trial, as well as data reported by the participants in the participant questionnaire at 7 days, 35 days and 4 months post randomisation. A second, broader definition of infection will additionally include any infections resulting in hospitalisation as well as any infection not resulting in hospitalisation as reported through participant post-procedure questionnaire, *ProBE-General Symptoms* questionnaire or SAE forms and measured at 7 days, 35 days and 4 months post randomisation.

The number of *subsequent prostate biopsy procedures* will be collected using the participant questionnaire at 35 days and 4 months post randomisation and reported by clinicians in the 4 months post-biopsy case report form.

*Burden and rate of detection of clinically insignificant PCa* will be reported by clinicians in the histology report at 7 days post randomisation. Clinically insignificant PCa will be defined as GGG1 disease [6]. We will also report on the rate of detection of GGG ≥ 3 PCa as an alternative definition of clinically significant disease.

### Sample size

The target sample size is 1,042 randomised participants (521 per group). This assumes a detection rate of clinically significant PCa in previously biopsy naïve individuals through TRUS biopsy following a pre-biopsy MRI of 45%, in line with the reported literature and data collected from 792 patients in Oxford over a 12-month period [13]. We consider an improvement of 10% (i.e. from 45 to 55% from TRUS to LATP) in the rate of detection of clinically significant PCa (defined GGG ≥ 2, i.e. any Gleason pattern ≥ 4 disease) through LATP to be a clinically significant primary outcome difference. This sample size assumes 90% power and 5% significance to detect a statistically significant difference between the two biopsy procedures in the primary outcome. No loss to follow-up was anticipated, as it was expected that all participants recruited and randomised would proceed to biopsy and PCa detection results would be available for all biopsies.

### Statistical analysis

#### General analysis principles

We will carry out all analyses on the intention-to-treat population (ITT) (i.e. participants will be analysed in the group they were randomised to regardless of actual biopsy received). We do not anticipate significant numbers of protocol deviations, but if any occur, we will repeat the primary analysis in the Per Protocol (PP) population. The PP population is a subset of the ITT population and will exclude participants with major protocol deviations (e.g. due to clinician decision, participant preference, or withdrawal), or where the participant did not satisfy the eligibility criteria, or where the biopsy was not conducted in accordance with the protocol as reported by sites (e.g. greater than or fewer than the required range of biopsy core numbers).

Standard descriptive statistics will be used to describe the participant baseline demographics for each of the two biopsy groups, reporting means and standard deviations (SD) or medians and interquartile ranges (IQR) as appropriate for continuous variables and numbers and percentages for binary and categorical variables. Analyses will be conducted using Stata (StataCorp LP, www.stata.com) or other well-validated statistical software.

#### Statistical interim analyses

No formal interim analyses of the primary or key secondary outcomes with stopping guidelines are planned. An independent DSMC will review the accumulating data at regular intervals and may recommend pausing or stopping the trial in the event of safety concerns.

The first stage internal pilot will have a formal ‘stop/go’ review of the randomisation numbers after 6 months of recruitment to the RCT. If the target of at least 140 randomisations has been met, the trial will continue to recruit for a further 9 months. Data from these 140 participants will be included in the final analysis alongside data from the study participants recruited once the pilot period ends.

#### Statistical significance and multiple testing

All comparative outcomes will be presented as suitable measures of intervention effect and reported together with 95% confidence intervals (CIs), and all tests will be carried out at a 5% two-sided significance level. The trial is powered for a single pre-specified primary endpoint and pre-specified analysis plan, and therefore no adjustment for multiple testing will be conducted.

#### Description of study patient throughput

**Fig. 1:**
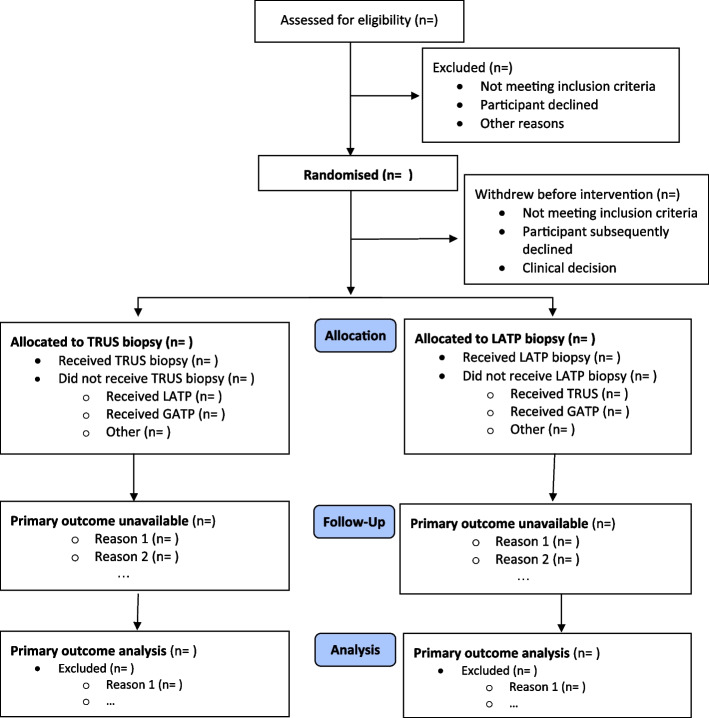
CONSORT flow chart. Some patients may have more than one reason for exclusion

The flow of participants through each stage of the trial, including number of participants screened, numbers eligible, numbers and reasons for ineligibility, numbers giving their consent and randomised, receiving the allocated intervention, and analysed for the primary outcome analysis will be provided using a CONSORT PRO extension flow chart [14] as per Fig. 1.

#### Withdrawal from intervention and/or follow-up or deaths

The number and percentage of participants withdrawn from the trial will be summarised by intervention group, with an indication of whether the withdrawal was before or after the biopsy procedure. In addition, details on the average time (in days) to withdrawal from randomisation and the reasons for withdrawal will also be summarised, where known. If any deaths occur during the study, the number and percentage as well as the time from biopsy to death (in days) will also be summarised.

#### Missing data

The number and percentage of participants with available data for each outcome at each time point will be summarised by intervention group. Patterns of missingness will be investigated, and the suitability of missing data assumptions considered. Reasons for data missingness, if known, will be explored and described.

#### Approach to missing data in analyses

The main analyses will be performed on an available case basis. For the primary outcome, the effect of missing data will be investigated, and multiple imputation (MI) used if more than 10% of primary outcome data is missing under a missing at random (MAR) assumption. For outcomes measured at multiple time points and analysed using repeated measures mixed effects models, unavailable observations either due to missed visits or due to a participant leaving the trial early are assumed to be similar to observed outcomes from similar participants at the same time points under a MAR assumption [15]. We do not anticipate using MI for missing outcome data in the repeated measures analysis, as MI also assumes a MAR mechanism and is therefore not expected to add value to the analysis model. If the data appear to be missing not at random, then alternative approaches, such as the *rctmiss* command in Stata [16] (or an equivalent if another statistical package is used) will be utilised to assess the impact of the assumptions on the estimated intervention effect.

#### Description of compliance with intervention

This trial will compare two methods that obtain prostate biopsies to detect clinically significant PCa (i.e. cases of PCa that are likely to require treatment), namely TRUS versus LATP. Intervention compliance in this trial is defined as the participant receiving the biopsy procedure to which they were randomised. Data on biopsy received will be summarised together with reasons why the allocated biopsy was not administered to the participant where this is relevant. The impact of non-compliance with the allocated intervention will be explored as part of the PP population analysis (as described in the “Analysis methods” section below).

#### Analysis methods

##### Analysis of primary outcome

The primary outcome is the difference in the detection rate of clinically significant PCa upon pathology reporting of biopsy samples between the TRUS and LATP biopsy groups, the clinically significant disease being defined as a GGG (ISUP) score of 2 or more, i.e., any Gleason Pattern ≥ 4 disease [6]. Detection rates for the two biopsy intervention groups will be compared using a mixed effects logistic regression model adjusted for the minimisation factors (recruitment centre, and site of prostatic lesion on pre-biopsy MRI as “no significant lesion” versus “significant lesion including anterior” versus “significant lesion, but not anterior”). Recruitment centre will be fitted in the model as a random effect to account for any heterogeneity due to the centre, and the radiologist-confirmed MRI pre-biopsy site of the prostatic lesion will be fitted as a fixed effect. The adjusted difference in detection rate of clinically significant PCa between the two groups will be presented as an Odds Ratio (OR), along with its associated 95% CIs. The primary analysis of the primary outcome will be conducted for the ITT population using available cases and will be repeated in the PP population.

#### Supporting analysis

As supporting analyses, we will conduct an unadjusted analysis, and a further analysis adjusting for additional important prognostic factors measured before biopsy. These additional model covariates will include age (years) as a continuous variable, PSA level (ng/ml) as a continuous variable, MRI tumour stage (using three categories: 1c [no lesion]; 2a, 2b, 2c [localised PCa]; 3a, 3b, 4 [locally advanced PCa]) as categorical variables, and cancer risk group (using two categories: breast or PCa family history; no PCa history) as categorical variables. The proportion of participants in each randomised group with positive and negative biopsy results will be tabulated, and the difference between groups reported as ORs and absolute differences together with 95% CIs.

##### Analysis of secondary outcomes

All secondary outcomes will be assessed by the intervention group for the ITT population. Raw means and SDs will be reported for continuous outcomes and counts with percentages for binary ones. Continuous outcomes will be analysed using mixed effects linear regression, and logistic regression will be used to analyse binary data. Multilevel models will be used for variables measured at multiple time points (I-PSS, IIEF, EQ-5D-5L) to allow for repeated measures clustered within each participant. All secondary outcome models will be adjusted for baseline measurement where possible and for the following minimisation factors: recruitment site (as a random effect) and radiographer-confirmed MRI lesion location (as a fixed effect). An intervention by time point (as a categorical variable) interaction will be included in the model, indicating the follow-up time point to which the outcome refers. The intervention effect at each time point, as well as the overall effect across time points, will be presented as ORs or mean difference (with SDs), depending on outcome. Model assumptions, including approximate normality of the residuals, will be assessed as relevant. All secondary outcome analyses will use two-sided 5% significance and 95% CIs with associated *p*-values reported throughout.

#### ProBE – Perception and ProBE – General Symptoms

*The ProBE – Perception* data collected immediately after the biopsy procedure will assess the between-group difference in the proportion of participants reporting a moderate or major problem in one or more items. The *ProBE – General Symptoms* part collected at 7 days post-biopsy will assess the between-group difference in the proportion of participants reporting a moderate or major problem in one or more complications [12]. Models to analyse this data will use mixed effects logistic regression and will be adjusted for the minimisation factors (recruitment centre and lesion location) as described above.

#### Infection

Infection rates will be assessed and compared between the two biopsy groups using the primary and secondary definitions of infection. The number of participants with at least one infection following biopsy will be compared between the two groups using a mixed effects logistic regression model adjusted for the stratification factors recruitment site (as a random effect) and lesion location (as a fixed effect).

#### Complications and subsequent biopsy procedures

The number and percentage of participants experiencing each type of biopsy-related complication, and those experiencing a biopsy-related complication at least once (such as urinary retention, blood in bowel movements, urology admission due to urinary bleeding, and urology admission due to pain), will be presented by intervention group and compared. Biopsy-related complications such as reduced sexual or urinary function will be reported as part of the secondary outcome IIEF [7]. The number of participants undergoing a subsequent biopsy procedure over the 4-month trial follow-up period and details of the subsequent biopsies will be summarised by intervention group.

#### Histology parameters

Histological parameters (ISUP grade group, cancer core length, core involvement, target biopsy cancer parameters) will be summarised descriptively by the intervention group and compared. An additional assessment of clinically significant PCa will be conducted based on a definition of GGG ≥ 3 (equivalent to Gleason Score 7 (4 + 3), Gleason Score 8 or Gleason Score 9–10) and compared between the two biopsy types. Participant burden rate will be described and evaluated based on the rate of clinically insignificant PCa detection (i.e. GGG score of 1).

#### Safety

It is anticipated that the rate of SAEs will be low in this trial. The total number of SAEs will be summarised by intervention group alongside the number and percentage of participants reporting at least one SAE. Details of the events, including the expectedness and relatedness of the SAEs, will be presented, together with information on the timing of the events. The proportion of participants with at least one SAE will be compared.

#### Pre-specified subgroup analysis

Subgroup analyses for the primary outcome are planned. We will assess whether the intervention effect differs between patient groups with differing MRI lesion status (recorded as ‘significant lesion including anterior’, ‘significant lesion not anterior’, or ‘no significant lesion’) and prostate volume (normal < 50 cc, large 50–79 cc, extra-large ≥ 80 cc). We hypothesise that the detection rate of clinically significant PCa will be higher in participants with an anterior lesion, and in those with a larger prostate, on MRI receiving an LATP versus TRUS biopsy [17]. Where numbers within subgroups are too small, we will consider combining levels within subgroups (e.g. combine prostate volume 50–79 cc with ≥ 80 cc). Subgroup analyses will use mixed effects logistic regression with a random effect for the recruitment site and a fixed effect for MRI pre-biopsy lesion location for the ITT population, in line with the analysis described for the primary outcome. Intervention allocation by subgroup interaction terms will be used to assess point estimates and confidence intervals and will be displayed using forest plots.

#### Sensitivity analyses

Sensitivity analyses will be conducted to assess the robustness of the main trial results. Sensitivity analyses to explore the impact of missing primary outcome PCa detection are outlined in the “Approach to missing data” in the “Analyses” section.

### Supplementary analyses and outcomes

#### Number of biopsy cores

The number of recommended biopsy cores to be taken for each type of intervention is stipulated in the protocol such that it is expected to be similar across the two biopsy groups. The number and proportion of participants with too many (more than 18 systematic cores) or too few (less than 6 systematic cores) will be presented for each randomised group.

#### Bayesian re-design methodological add-on

A methodological Bayesian re-design of the trial data was pre-specified in the study protocol and is being conducted alongside the main trial [3]. This re-design intends to assess whether analysing TRANSLATE using novel Bayesian adaptive methods may provide efficiency gains over the more typical trial design outlined in this SAP. The final reporting of trial results will be presented in line with the aspects described above and will be considered independent of the methodological outcomes. The Bayesian final analysis will be conducted once the study has completed data collection and will be presented after the trial primary results have been reported. Details of the Bayesian methodology employed are described in the Additional file Appendix 1.

### Statistical packages

All analysis will be carried out using appropriate validated statistical software such as Stata v18.0 (StataCorp, College Station, TX, USA), SAS 9.4 (SAS Institute, Cary NC, USA) or R v3.4.1 (R Foundation for Statistical Computing, Vienna, Austria). The relevant package(s) and version number(s) will be recorded in the Statistical Report.

## Discussion

This update to the TRANSLATE trial protocol contains the pre-specified SAP manuscript written according to the published guidelines on the content of SAPs [2]. The publication of the SAP aims to increase the transparency of the data analysis and reduce the risk of outcome reporting bias. Any deviations or changes to the current SAP will be described and justified in the final study report and results publication.

## Trial status

Recruitment into the trial opened on 3rd December 2021. Follow-up for the trial is ongoing and will be completed by March 2024, followed by the outcomes analysis.

### Supplementary Information

Additional file 1: Appendix 1.

## Data Availability

Not applicable.

## Abbreviations

CI: Confidence intervals
CONSORT: Consolidated Standards of Reporting Trials
CRF: Case report form
DRE: Digital rectal examination
DSMC: Data and Safety Monitoring Committee
eGFR: Estimated glomerular filtration rate
GCP: Good Clinical Practice
GGG: Gleason Grade Group
IIEF: International Index of Erectile Function
I-PSS: International Prostate Symptom Score
IQR: Interquartile range
ITT: Intention-to-treat
MAR: Missing at random
MI: Multiple imputation
MRI: Magnetic resonance imaging
LATP: Local anaesthetic transperineal
OCTRU: Oxford Clinical Trials Research Unit
OR: Odds ratio
PCa: Prostate cancer
ProBE: Prostate Biopsy Effects (Questionnaire)
PP: Per protocol
PSA: Prostate-specific antigen
RCT: Randomised controlled trial
SAE: Serious adverse event
SAP: Statistical analysis plan
SD: Standard deviation
TRANSLATE: TRANSrectal biopsy versus Local Anaesthetic TransPerineal biopsy Evaluation
TRUS: Transrectal ultrasound-guided
TSC: Trial Steering Committee
VAS: Visual analogue scale

## References

[1] Scattoni V, Zlotta A, Montironi R, Schulman C, Rigatti P, Montorsi F (2007). Extended and saturation prostatic biopsy in the diagnosis and characterisation of prostate cancer: a critical analysis of the literature. Eur Urol.

[2] Gamble C, Krishan A, Stocken D, Lewis S, Juszczak E, Doré C (2017). Guidelines for the content of statistical analysis plans in clinical trials. JAMA - Journal of the American Medical Association.

[3] Bryant RJ, Yamamoto H, Eddy B, Kommu S, Narahari K, Omer A (2023). Protocol for the TRANSLATE prospective, multicentre, randomised clinical trial of prostate biopsy technique. BJU Int..

[4] Harris PA, Taylor R, Thielke R, Payne J, Gonzalez N, Conde JG (2009). Research electronic data capture (REDCap)—a metadata-driven methodology and workflow process for providing translational research informatics support. J Biomed Inform.

[5] Harris PA, Taylor R, Minor BL, Elliott V, Fernandez M, O’Neal L (2019). The REDCap consortium: building an international community of software platform partners. J Biomed Inform.

[6] Prostate Cancer Foundation (Institution) IS of UP. Gleason Score and Grade Group. 2014 Available from: https://www.pcf.org/about-prostate-cancer/diagnosis-staging-prostate-cancer/gleason-score-isup-grade/ Cited 2021 Sep 22.

[7] Rosen RC, Riley A, Wagner G, Osterloh IH, Kirkpatrick J, Mishra A (1997). The international index of erectile function (IIEF): a multidimensional scale for assessment of erectile dysfunction. Urology.

[8] Barry MJ, Fowler FJ, O’leary MP, Bruskewitz RC, Holtgrewe HL, Mebust WK (2017). The American Urological Association Symptom Index for Benign Prostatic Hyperplasia. J Urol.

[9] Brooks R (1996). EuroQol: the current state of play. Health Policy (New York).

[10] Herdman M, Gudex C, Lloyd A, Janssen M, Kind P, Parkin D (2011). Development and preliminary testing of the new five-level version of EQ-5D (EQ-5D-5L). Qual Life Res.

[11] Hernández-Alava M, Pudney S (2018). eq5dmap: A command for mapping between EQ-5D-3L and EQ-5D-5L. Stata Journal..

[12] Mian BM, Kaufman RP, Fisher HAG (2021). Rationale and protocol for randomized study of transrectal and transperineal prostate biopsy efficacy and complications (ProBE-PC study). Prostate Cancer Prostatic Dis.

[13] Bryant RJ, Hobbs CP, Eyre KS, Davies LC, Sullivan ME, Shields W (2019). Comparison of Prostate Biopsy with or without Prebiopsy Multiparametric Magnetic Resonance Imaging for Prostate Cancer Detection: An Observational Cohort Study. J Urol.

[14] Calvert M, Blazeby J, Altman DG, Revicki DA, Moher D, Brundage MD (2013). Reporting of patient-reported outcomes in randomized trials: The CONSORT PRO extension. JAMA - Journal of the American Medical Association..

[15] Sullivan TR, White IR, Salter AB, Ryan P, Lee KJ (2018). Should multiple imputation be the method of choice for handling missing data in randomized trials?. Stat Methods Med Res.

[16] White I (2017). Stata module to analyse a randomised controlled trial (RCT) allowing for informatively missing outcome data. Computer Science Statistical Software Components.

[17] Omer A, Lamb AD (2019). Optimizing prostate biopsy techniques. Curr Opin Urol.

