# Supplementary material for: Statistical analysis plan for the TRANSLATE (TRANSrectal biopsy versus Local Anaesthetic Transperineal biopsy Evaluation of potentially clinically significant prostate cancer) multicentre randomised controlled trial
Source: Trials. 2024 Jun 14;25:383. doi: 10.1186/s13063-024-08224-4 (PMC11177457; doi:10.1186/s13063-024-08224-4)
Supplement: Supplementary file 1 — Additional file 1: Appendix 1. [file 13063_2024_8224_MOESM1_ESM.docx]

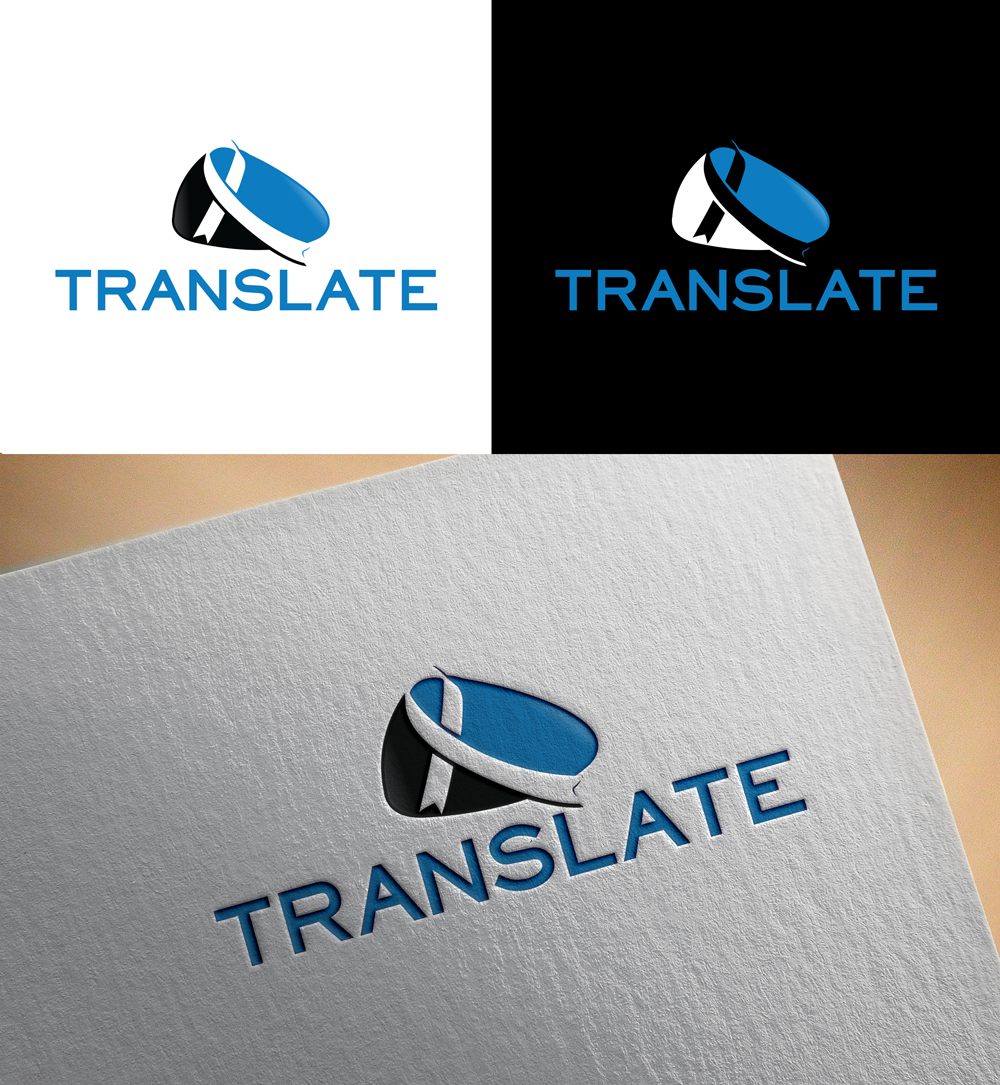


**Study Title: A randomised controlled trial of TRANSrectal biopsy versus Local Anaesthetic Transperineal biopsy Evaluation (TRANSLATE) of potentially clinically significant prostate cancer**

**Bayesian Analysis Plan (BAP)**

Version 1.0 - 31Jan2024

Trial Registration: ISRCTN 98159689

Based on Protocol Version 3.0 – 14Nov2022

**Oxford Clinical Trials Research Unit (OCTRU)**

**and**

**Centre for Statistics in Medicine (CSM)**

**
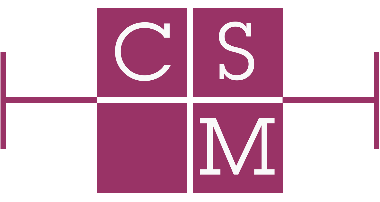

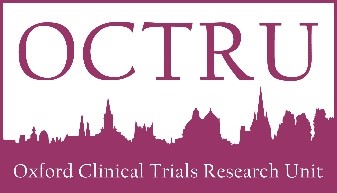
**

Contents

[Key personnel 2](#_Toc167099065)

[Background 4](#_Toc167099066)

[Statistical Analysis Outline 4](#_Toc167099067)

[Simulation Study (Trial Design) 5](#_Toc167099068)

[Trial Re-Analysis 7](#_Toc167099069)

[Appendix: TRANSLATE Bayesian Equations 8](#_Toc167099070)

[1. Conjugate Simulation Study 8](#_Toc167099071)

[2. Logistic Regression Trial Analysis 9](#_Toc167099072)

[References 11](#_Toc167099073)

#

# Key personnel

**Author(s) (Trial statistician(s))**

Alexander Ooms [alexander.ooms@ndorms.ox.ac.uk](mailto:alexander.ooms@ndorms.ox.ac.uk) (OCTRU Senior Statistician)

Ioana Marian [ioana.marian@csm.ox.ac.uk](mailto:ioana.marian@csm.ox.ac.uk) (TRANSLATE Senior Statistician)

**Reviewers (Chief Investigators)**

Assoc. Prof Richard John Bryant [richard.bryant@nds.ox.ac.uk](mailto:richard.bryant@nds.ox.ac.uk) (Co-Chief Investigator)

Mr. Alastair Lamb [alastair.lamb@nds.ox.ac.uk](mailto:alastair.lamb@nds.ox.ac.uk) (Co-Chief Investigator)

Jane Holmes [jane.holmes@phc.ox.ac.uk](mailto:jane.holmes@phc.ox.ac.uk) (Former TRANSLATE Senior Statistician)

Matthew Parkes [matthew.parkes@manchester.ac.uk](mailto:matthew.parkes@manchester.ac.uk) (Former TRANSLATE Senior Statistician)

# Background

The TRANSLATE trial is a phase III, UK-wide, multicentre, superiority, 1:1 randomised clinical trial that aims to provide a robust evaluation of the performance characteristics of two biopsy procedures in biopsy-naïve men being investigated for possible clinically significant prostate Cancer (PCa). The study aims to recruit biopsy-naïve men referred with suspected PCa based on an elevated age-specific Prostate Specific Antigen (PSA) or abnormal digital rectal examination (DRE), and suitable for investigation with pre-biopsy MRI and prostate biopsy.

**Trial interventions**

Men eligible for the study with a pre-biopsy MRI scan as part of the investigation for possible prostate cancer (PCa) will be consented and randomised to receive either:

1. TRUS (transrectal) biopsy
2. LATP (local anaesthetic transperineal) biopsy

**Primary outcome**

The primary outcome in TRANSLATE is the difference in detection rate of clinically significant PCa upon pathology reporting of biopsy samples between the TRUS and LATP biopsy groups. Clinically significant PCa is defined in this trial as Gleason Grade Group ≥2, i.e. any Gleason pattern ≥4 disease. This is a pathology-based endpoint, usually available within 7 days of the initial biopsy having been undertaken, but difficult cases or other pathway delays may result in a longer period of time being taken.

**Sample Size**

Data collected from 792 patients in Oxford over a 12-month period suggests that the detection rate of clinically significant PCa in previously biopsy naïve individuals through TRUS biopsy following a pre-biopsy MRI is 45% (14), in line with the reported literature. We consider a 10% improvement (from 45% to 55%) in this rate of detection of clinically significant PCa (defined as Gleason Grade Group ≥2, i.e. any Gleason pattern ≥4 disease) through LATP to be clinically meaningful. To detect this primary outcome difference with 90% power and 5% significance, we need to recruit 1,042 men over a 15-month period across a minimum of nine participating centres. Full details of the TRANSLATE trial protocol have been published previously [1]

# Statistical Analysis Outline

As a ‘methodological add-on’ to the main TRANSLATE trial, we will re-design the trial using an adaptive Bayesian methodology to explore the relative efficiency savings of running the trial had it been designed as an adaptive Bayesian trial with planned interim analyses with early stopping rules, rather than the fixed trial with no early stopping and frequentist analysis which forms the *actual* *real-world* primary outcome analysis (see the Statistical Analysis Plan (SAP) for full details). We will initially use noninformative priors for the Bayesian trial, and may also consider informative priors (elicited from experts and/or data). We will use simulation to decide on the number and timing of interim analyses and the thresholds for decision boundaries of the early stopping rules. Once the trial has finished, we will use this adaptive Bayesian design with the trial data as it accrued to see what would have happened had the trial been run with the Bayesian design, for example if the trial would have stopped early for futility or superiority and compare the final result with that of the actual trial primary analysis. If the trial would have stopped early, then the Bayesian final analysis will be on a reduced sample size than that of the actual trial.

# Simulation Study (Trial Design)

A simulation study will be performed to assess operating characteristics and finalise parameters to be used in the trial re-design. A full description of the methods used in this simulation study is given in Appendix: TRANSLATE Bayesian Equations.

**Aim**

The aim of the simulation study is to produce a final Bayesian trial re-design of the TRANSLATE trial, specifying the number of interim analyses, their timings and early stopping criteria.

**Data-generating mechanisms**

The detection rate for each group, will be generated from independent Binomial distributions based on the statistical principles underlying the TRANSLATE trial design using R. The detection rate of clinically significant PCa (defined as Gleason Grade Group ≥2, i.e. any Gleason pattern ≥4 disease) through TRUS biopsy following a pre-biopsy MRI is assumed to be 45%. A 10% improvement from 45% to 55% through LATP versus TRUS in this rate of detection is considered clinically meaningful in TRANSLATE, using 90% power and 5% significance [1].

**Simulated trial results**

From the simulations the following measures will be obtained to evaluate each simulated scenario:

- Average trial size
- Minimum trial size
- % of trials stopping early for success, for futility and overall (all instances of stopping early for any reason)
- Simulated Type I Error
- Simulated Power.

Using these outputs, the final Bayesian trial will be determined. The parameters that will determine the final design are:

- Number of interim analyses
- Posterior probability of superiority threshold required to have “success” at the final analysis
- Posterior probability of superiority threshold required to stop early for success
- Posterior predictive probability of success threshold required for stopping early for futility.

In keeping with the design of the main study, the trial design that generates a one-sided type I error rate of 2.5% (equivalent to two-sided 5% error rate) and 90% power will be used for the Bayesian trial.

**Methods**

Posterior predictive probabilities will be used to assess stopping for futility, and posterior probabilities will be used in stopping rules for success.

The analysis used in the simulation study will be unadjusted. Simulations will be run using conjugate Beta Binomial models to determine key trial parameters & thresholds.

Table 1 outlines all information used in the initial set of simulations. Further simulations may be run to finalise parameters including number of interim analyses; these further studies will not be described in this analysis plan. The initial stopping thresholds for early stopping/ superiority presented in Table 1 have been chosen arbitrarily and will likely be refined during further iterations of this simulation study (details of further iterations of the simulation study will not be included in this document). The initial number of interim analyses given in Table 1 (0 or 1) have been chosen for computational reasons. All thresholds will be explored and refined first based on one or no interim analyses, and once those thresholds have been (approximately) found, the impact of additional interim analyses will be explored. It is likely that the final Bayesian adaptive trial design will feature more than one interim analysis.

Table 1 Initial Simulation Study Parameters

| **Item** | **Fixed/ Varied** | **Value (if fixed)/**  **Scenarios (if varied)** |
| --- | --- | --- |
| Number of simulations per scenario | Fixed | 1000 |
| Maximum trial size | Fixed | 1042 (identical to TRANSLATE main study) |
| Priors on detection rate for TRUS & LATP | Fixed | Beta(1,1) |
| Posterior probability of superiority (LATP > TRUS) required to stop for success (interim) | Varied | 0.98, 0.99 |
| Posterior probability of superiority (LATP > TRUS) required to stop for success (final) | Varied | 0.95, 0.98 |
| True detection rates | Varied | Four “truth” scenarios*:   - Null - Power - Half - Double   Null:  TRUS detection rate=0.45  LATP detection rate=0.45  Power:  TRUS detection rate=0.45  LATP detection rate= 0.55  Half:  TRUS detection rate=0.45  LATP detection rate= 0.5  Double:  TRUS detection rate=0.45  LATP detection rate= 0.65 |
| Number of interim looks | Varied | 0, 1  Timing of the interim looks will be spaced evenly through the sample size i.e.   - one look at 600 participants (>50% of target sample size) |
| Posterior predictive probability of success threshold required for stopping for futility | Varied | 0.05, 0.1 |

** Null scenario gives overall cancer detection rates of 45% in both groups (null hypothesis). Power scenario gives the detection rates the original trial is designed to observe; 45% detection rate in TRUS and 55% detection rate in LATP (θ=0.1). Half scenario gives half the detection rates the original trial is designed to observe; 45% detection rate in TRUS and 50% detection rate in LATP (θ=0.05). Double scenario gives double the detection rates the original trial is designed to observe; 45% detection rate in TRUS and 65% detection rate in LATP (θ=0.2).*

Simulation study results, including finalised trial design, will be written up and presented as supplemental material to the Bayesian results publication. These results will be date stamped to show the trial design was finalised prior to the final study data being available.

# Trial Re-Analysis

**Aim**

To perform a Bayesian analysis of the primary outcome of TRANSLATE, including pre-specified formal interim analyses with the possibility of stopping early for success (efficacy) or futility. If possible, the re-analysis will be performed in “real-time”, i.e. in parallel with, or soon after, the main frequentist analysis has been completed.

**Outcome**

The primary outcome of the TRANSLATE RCT is “to compare the TRUS biopsy versus LATP biopsy in the detection of clinically significant PCa (defined as Gleason Grade Group ≥2, i.e. any Gleason pattern ≥4 disease)”. This pathology-based endpoint is usually available within seven days of the biopsy being taken.

**Analysis Population**

The analysis population will match that described in the main trial’s SAP and will follow the intention-to-treat principle. Further details of how withdrawals, treatment crossovers, etc. are given in detail in the main SAP. If the trial would have stopped early under the adaptive Bayesian design, the analysis population will be smaller than that of the actual trial analysis population but will follow the same definition for inclusion.

In addition, if the Bayesian trial is smaller than TRANSLATE, we will run the Bayesian analysis over different samples of the actual trial data to assess robustness of the results. Samples will be drawn from the full dataset with replacement.

**Methods**

Numbers in each treatment group, and PCa detection proportions, will be presented. The primary outcome will be compared between the two randomised groups using logistic regression. The regression model will match that described for the primary analysis in the main trial SAP, including what factors are adjusted for and is described fully in Appendix: TRANSLATE Bayesian Equations. Posterior estimates and 95% credible intervals of parameters of interest will be reported. Direct probability statements based on the posterior distributions, e.g. the probability of observing an improvement in the detection rate, will be produced.

Analysis methods at interim analyses will be the same as those used for the final analysis.

An unadjusted supporting analysis will be performed using a conjugate Beta Binomial model at each planned analysis. Beta (1,1) priors will be used for these analyses.

**Stopping criteria**

Stopping early for success will be based on direct posterior probabilities of superiority (one-way: LATP > TRUS), the threshold of probability of superiority required to stop early is determined using methods described in the Simulation Study section.

Early stopping for futility will be determined through posterior predictive probabilities of success (PPOS), with success again determined one-way LATP > TRUS. The PPOS will be calculated at each interim analysis. If the PPOS falls below a specified threshold (determined via simulation) at any interim analysis, the trial will stop for futility.

**Statistical packages**

Analysis will be carried in out in R. JAGS, OpenBUGS (MCMC) or Stan (HMC) will be used to sample from target posterior distributions and accessed through R packages (e.g. R2OpenBUGS [2], rjags, RStan [3]). Simulation study will be carried out using the same packages.

# Appendix: TRANSLATE Bayesian Equations

Please note some abuses in notation both in subscripts and conditional probabilities. These have been omitted for readability.

## Conjugate Simulation Study

We will perform a simulation study over many different trial designs using conjugate beta distributions for computational efficiency to identify a target trial design that satisfies our restrictions (e.g. type I error & power).

For group *j* at interim *k,* let $n_{jk}$ be the number of participants recruited, $y_{jk}$ the number of participants with PCa detected, and $\theta_{j}$ the probability of response*.* Then

$$\theta_{j}\sim\text{ Beta}\left( 1, 1 \right), j=1,2$$

The posterior distribution for $\theta_{j}$ at interim *k* is given by

$$\theta_{j}|\mathrm{Data}_{\mathrm{jk}} \sim\text{Beta}(1+ y_{jk}, 1+n_{jk}- y_{jk})$$

where $\mathrm{Data}_{\mathrm{jk}}$ is the observed data at interim *k* for group *j.*

The posterior probability that LATP has a higher detection rate than TRUS is given by $P(\theta_{1}>\theta_{2}|\text{Data)}$. This can be rewritten as $P\left( \theta_{1}- \theta_{2}>0 \right|\text{D}ata)$ which can be approximated by a normal distribution [4].

**Superiority**

If the trial does not stop early, LATP will be declared superior to TRUS if the posterior probability of superiority is greater than a predefined threshold, $\eta_{final}$, i.e. if

$$P\left( \theta_{1}-\theta_{2}>0 | Data_{final} \right)> \eta_{final}$$

The threshold $\eta_{final}\in\left[ 0,1 \right]$ is a constant to be determined via simulations.

**Early stopping rules**

*Superiority*

At interim *k*, if $Data_{k}$ denotes the data observed so far, LATP will be declared superior to TRUS if

$$P\left( \theta_{1}-\theta_{2}>0 | Data_{k} \right)> \eta_{int}$$

where $\eta_{int}\geq\eta_{final}$ and is determined via simulations.

*Futility*

At interim *k,* we will stop the trial for futility if the Posterior Predictive Probability of Success (PPOS) is less than a predetermined threshold γ. This means we will only continue if there is a high chance of claiming success if we were to continue to recruit to the maximum sample size.

At interim *k,* the posterior predictive distribution is Beta-Binomial. In group *j* if $n_{jk}$ participants have been recruited so far, then there are $m_{jk}=N_{j,max}-n_{jk}$ participants left to recruit. Let $\tilde{y}_{j}$ denote new responses in future patients, then the posterior predictive distribution is given by:

To calculate the PPOS at interim analysis $k$, iterate the following steps many times:

1. For each group *j*, randomly generate new outcome data $\tilde{y}_{j}$ from the relevant posterior predictive distributions.
2. Using this now “complete” dataset the posterior distribution for $\theta_{j}$ is given by:

$$\theta_{j} \sim Beta(1+y_{jk}+ \tilde{y}_{j}, 1+N_{j,max}-y_{jk}-\tilde{y}_{j})$$

3. Check if $\mathbb{P}\left( \theta_{1}-\theta_{2}>0|Data \right)>\eta_{final}$ for this iteration of randomly sampled “new” data.
4. Record the result of step 3 (superiority found/ superiority not found).

The PPOS at interim analysis $k$ is given by:

$$PPOS=\frac{\text{number of times} \mathbb{P}\left( \theta_{1}-\theta_{2}>1|Data \right)>\eta_{final}}{\text{total number of iterations}}.$$

If $PPOS<\gamma$, we will stop the trial for futility at interim *k*. $\gamma\in\left[ 0,1 \right]$ is a constant to be determined via simulations.

## Logistic Regression Trial Analysis

The primary analysis model for the TRANSLATE trial is a frequentist mixed effects logistic regression. This will be replicated in a Bayesian framework. The model form, priors and stopping rules will be described in this section. Posterior estimates will be obtained via MCMC methods using a resampling program such as BUGS or Stan.

The regression equation used is:

$$logit\left( \theta_{i,s} \right)=\beta_{0}+\beta_{1}*treatment_{i}+\beta_{2}*lesion.location1_{i}+\beta_{3}*lesion.location2_{i}+b_{i}$$

Where:

- $treatment_{i}=1$ if LATP, $0$ if TRUS.
- $lesion.location1_{i}=1$ if significant lesion including anterior, $0$ if no significant lesion
- $lesion.location2_{i}=1$ if significant lesion, but not anterior, $0$ if no significant lesion
- $b_{i}\sim N\left( 0,\sigma_{b}^{2} \right)$ represents the between site effect and $\sigma_{b}^{2}=$ between site variance

**Priors:**

$\beta_{i}\sim N\left( 0,{2.5}^{2} \right), i=\left( 0,1,2,3 \right)$

- $\sigma_{b}\sim Half-normal\left( 0, {2.5}^{2} \right)$ [5]

Baseline covariates will be standardised according to Gelman [6]so that dichotomous covariates have mean zero and differ by 1 in their upper and lower condition.

**Superiority**

We say LATP is superior to TRUS if, at the final analysis:

$$\mathbb{P}\left( \beta_{1}>1|Data_{final} \right)>\eta_{final}.$$

Similarly, we say LATP is superior to TRUS if, at interim analysis $k$:

$$\mathbb{P}\left( \beta_{1}>1|Data_{k} \right)>\eta_{int}.$$

**Futility**

Baseline characteristics for predicted participants at interim analysis $k$ will be sampled with replacement from the current data with both treatment groups combined. Response data, $\tilde{y}$, will be predicted based on generated baseline characteristics and randomised group. $\beta_{1}$ will be calculated for this “complete” dataset via MCMC methods described above and the result (if $\mathbb{P}\left( \beta_{1}>1|Data \right)>\eta_{final}$) will be stored. This process will be iterated and the PPOS at interim $k$ will be defined as

$$PPOS=\frac{number of times \mathbb{P}\left( \beta_{1}>1|Data \right)>\eta_{final}}{total number of iterations}$$

the trial will stop for futility if, at interim analysis $k$:

$$PPOS<\gamma.$$

# References

[1] R. J. Bryant *et al.*, “Protocol for the TRANSLATE prospective, multicentre, randomised clinical trial of prostate biopsy technique,” *BJU Int*, 2023, doi: 10.1111/bju.15978.

[2] S. Sturtz, U. Ligges, and A. Gelman, “R2OpenBUGS: A Package for Running OpenBUGS from R.” Apr. 03, 2020.

[3] Stan Development Team, “RStan: the R interface to Stan.” 2024.

[4] John D. Cook, “Fast approximation of Beta inequalities ,” MD Anderson Cancer Center, 2012.

[5] A. Gelman, “Prior distributions for variance parameters in hierarchical models (comment on article by Browne and Draper),” *Bayesian Anal*, vol. 1, no. 3, Sep. 2006, doi: 10.1214/06-BA117A.

[6] A. Gelman, A. Jakulin, M. G. Pittau, and Y.-S. Su, “A weakly informative default prior distribution for logistic and other regression models,” *Ann Appl Stat*, vol. 2, no. 4, Dec. 2008, doi: 10.1214/08-AOAS191.
